# The Effect of Adding Paraffin Bath to the Therapeutic Hand Exercise Programme on Hand Functions in Patients with Systemic Sclerosis: A Randomized, Controlled, Single-Blind Study

**DOI:** 10.5152/ArchRheumatol.2026.25096

**Published:** 2026-02-23

**Authors:** Salim Mısırcı, Ali Ekin, Burcu Yağız, Belkis Nihan Coşkun, Ediz Dalkılıç, Lale Altan, Yavuz Pehlivan

**Affiliations:** 1 Division of Rheumatology, Department of Internal Medicine, Bursa Uludağ University Faculty of Medicine, Bursa, Türkiye; 2 Department of Physical Medicine and Rehabilitation, Bursa Uludağ University Faculty of Medicine, Bursa, Türkiye

**Keywords:** Exercise, non-pharmacological treatment, paraffin bath, systemic sclerosis

## Abstract

**Background/Aims:**

Paraffin bath therapy is used as a topical treatment for inflammatory and non-inflammatory diseases of the hands. The effects of adding paraffin bath therapy to the exercise programme on pain, hand movements, and hand function in patients with systemic sclerosis (SSc) and hand involvement were investigated.

**Materials and Methods:**

The study included 40 patients (a total of 245 patients were screened for eligibility) with hand dysfunction (Cochin Hand Functional Scale (COCHIN) ≥ 10). The patients were randomized into a control group (exercise only) (n = 20) and a paraffin bath group (exercise and paraffin bath) (n = 20). Patients were assessed with COCHIN, Modified Hand Mobility in Scleroderma (HAMIS), Delta Finger-To-Palm (DFTP), Hand Grip Strength (HGS), and Numerical Rating Scale (NRS) at baseline, 6 and 12 weeks after treatment.

**Results:**

Thirty-five patients (control group (n = 18), paraffin bath group (n = 17)) who had completed the study period were evaluated. There was a significant difference in the change values of the parameters COCHIN, HAMIS-Right Hand, HAMIS-Left Hand, HGS-Right Hand, and HGS-Left Hand at both the 6-week and 12-week follow-up examinations of the control and paraffin groups compared to the baseline values (*P* < .05 for all). In addition, there was a significant difference in the change values of the NRS and DFTP-Right Hand parameters in the paraffin bath group at both the 6-week and 12-week follow-ups, in addition to the existing parameters (*P* < .05 for both). When the 2 groups were compared with each other, there was a statistical difference in the NRS parameter in favor of the paraffin bath group (*P* = .007 in the 6th week, *P* < .001 in the 12th week). There was no difference between the groups for the other parameters (*P* > .05).

**Conclusion:**

Therapeutic hand exercises are effective for SSc patients with impaired hand function, and the addition of paraffin baths in the exercise program reduces pain.

**Main Points**

- In the course of systemic sclerosis (SSc), the functions of the hands may be impaired due to the involvement of the skin and joints.
- There are only a few studies investigating the effect of paraffin baths on the function of the hands of SSc patients.
- Therapeutic hand exercises are effective for SSc patients with impaired hand function, but the addition of a paraffin bath to the exercise program may be useful for pain relief, although it does not add any additional benefit to hand function.

## Introduction

Systemic sclerosis (SSc) is a multisystem autoimmune disease characterized by immune activation, vasculopathy, and fibrosis.^1^ Although lung involvement is the most common cause of morbidity, the skin is the most common site of involvement.^2^ In addition to skin involvement, various musculoskeletal symptoms such as arthralgia, arthritis, and tenosynovitis can also develop during the course of SSc.^3^ During the follow-up period, skin thickening, digital ulcers, and joint contractures may frequently occur on the hands, and the quality of life may be impaired.^4^

With new agents in the treatment of SSc, progress has been made in the prevention and treatment of complications such as skin and pulmonary fibrosis, pulmonary arterial hypertension, and digital ulcers. On the other hand, there is a need to standardize the use, content, and timing of non-pharmacological treatment modalities.^5^

In the non-pharmacological treatment of hand involvement, exercise applications are in the foreground due to their easy accessibility and low cost.^4^ Although studies have shown positive effects of exercise applications on hand function and quality of life in SSc patients, there is no standard exercise programme.^4^^,^^6^^-^^13^

Pain in the hands can occur in SSc patients due to various causes such as vasospasm, digital ulcer, synovitis, and joint contractures.^14^

Paraffin bath therapy can be used as a local treatment for both inflammatory and non-inflammatory diseases of the hands. The rise in tissue temperature in the treated area may cause hyperemia by relaxing the smooth muscle fibers in the arterioles, thus dilating the blood vessels. In the area where paraffin bath therapy is applied, the reduction in pain and muscle cramps, together with the increase in tissue viscoelasticity, may facilitate effective implementation of the exercise programme.^15^

In SSc patients, the use of paraffin baths may come to the fore, especially to relieve the pain caused by hand involvement and to facilitate the applicability of exercises that are important for hand movements. There are few studies on the efficacy of paraffin baths in patients with SSc, and the results are contradictory.^16^^-^^18^ Therefore, the effects of adding paraffin bath therapy to the exercise programme on pain, hand movements, and hand function in patients with SSc and hand involvement were investigated.

## Materials and Methods

### Study Design

This study was conducted as a single-blinded randomized controlled trial. Forty patients (a total of 245 patients were screened) who were eligible for the study were randomized into the control group (exercise only) (n = 20) and the paraffin bath group (exercise + paraffin bath) (n = 20) using a computer-assisted randomization program (https://www.random.org/). The randomization was conducted by someone not involved in the study. The permutation block method was used to ensure patients were equally distributed across the groups. In addition, all assessments were carried out by a single investigator who did not know which group the patients belonged to. This study was approved by the Ethics Committee of Bursa Uludağ University Faculty of Medicine (approval number: February 14, 2023, 2023-3/37).

### Patients Selection

Two hundred and forty-five patients with SSc diagnosed according to the 2013 American College of Rheumatology (ACR)/European League Against Rheumatism (EULAR) Classification Criteria^19^ who were followed up in the rheumatology clinic of a tertiary care hospital Bursa Uludağ University Faculty of Medicine were screened for eligibility. The inclusion criteria were being older than 18 years and hand dysfunction. The Cochin Hand Functional Scale (COCHIN) was used to determine hand dysfunction. Those with a COCHIN score ≥10 and those who agreed to participate in the study were included. When planning studies in SSc patients, it is recommended to include those with COCHIN scores of ≥10 or 15 to reduce the floor effect, taking into account the limits of initial disease activity.^20^ Therefore, patients were included in the study according to the COCHIN ≥10 criterion. Exclusion criteria were younger than 18 years, overlap with other connective tissue diseases, active digital ulcer, acute arthritis, upper limb amputation, treatment with hand exercises or paraffin baths in the last 3 months, allergy to paraffin, and systemic diseases that do not tolerate exercises or paraffin baths, diseases affecting cognitive function such as dementia, and inability to participate in the planned treatment program due to mobilization difficulties.

### Assessments

Written informed consent was obtained from all patients who agreed to take part at the beginning of the study. After the baseline assessment of the participants, both the control group and the group with the paraffin bath were instructed by a physiatrist in a therapeutic exercise program for the hands and received handouts. All patients followed the exercise program as a home program for 6 weeks and 3 days per week (30-45 minutes per day). Patients were asked to keep a diary to monitor adherence to the exercise program, and adherence to the exercises was checked by telephone every 2 weeks. The reason for conducting the exercise program at home was that it would be both cost and time efficient and would facilitate participation in the study as patients would feel more comfortable in their home environment.

The exercises for the hands consisted of therapeutic exercise movements from the exercise program used in the study by Santos et al.^21^ In the exercise program: Exercises with fabric for flexion and extension movements of the fingers (hold the fabric and release it), exercise with rubber ball (hold the ball and play in the basket); exercises with fabric and silicone finger apparatus for abduction and adduction movements of the fingers; exercises with fabric (grip the fabric by holding it with the thumb and every other finger), with small balls (try to hold the balls with the thumb and the other fingers), with a plastic bottle (try to close and open a previously opened plastic bottle cap); for wrist flexion and extension with a rubber ball (roll the rubber balls back and forth with the hand and wrist), with fabric (try to bend and fold the fabric); for resistance grip with a soft ball (grip the balls and apply force), a soft spring tool (hold the tool in the grip and apply force), an elastic hand helper (hold the tool in the grip and apply force). The indicated exercises were performed by the participants by doing 10 repetitions of each movement.

In the paraffin bath group, in addition to the exercise program, a paraffin bath application (3 days per week, 6 weeks) was performed with a physiotherapist in Bursa Uludağ University Faculty of Medicine Department of Physical Medicine and Rehabilitation.

For the application of the paraffin bath, the paraffin temperature was set to 45-55°C using a thermometer. The patient was instructed to dip the hand in and out of the paraffin bath 10 times with the wrist in a neutral position and the fingers open. The hand was then wrapped in a towel and kept for 20 minutes for heat preservation.

The evaluation in terms of possible side effects was determined by contacting the participants in the paraffin group when they came to the hospital for paraffin application and the exercise group by telephone.

The participants were assessed 3 times: at the beginning of the study, after completion of the 6-week treatment program, and 12 weeks after the start of the study to determine the long-term effects. The reasons for scheduling the study as a total of 12 weeks were that the duration of the study was consistent with the duration of previous studies, that patient compliance with the program is likely to decrease with longer follow-up periods, and that 12 weeks is a sufficient period for the benefits of paraffin bath application.

At the beginning of the study, the participants’ age, gender, duration of illness, and medical treatments were recorded. The Numerical Rating Scale (NRS) to assess wrist and hand pain, the COCHIN to assess hand functionality, the Modified Hand Mobility in Scleroderma (HAMIS) to assess hand movements, the Delta Finger-To-Palm (DFTP) to assess finger movements, and a dynamometer (TTM, DYNAMO METER TOKYO 100 kg) to assess hand grip strength were used.

The NRS is an assessment criterion for rapidly evaluating the severity of disease-related symptoms, categorizing and monitoring disease severity and control. It consists of progressively increasing numbers arranged on a line at equal intervals. The patient is asked to give a value between 0 and 10 for the pain they are experiencing (0 = no pain, 1-3 = mild pain, 4-6 = moderate pain, 7-10 = severe pain).^22^^,^^23^

The COCHIN consists of a scoring system ranging from 0 (no difficulty) to 5 (impossible) and includes 18 questions on daily activities with the hands. The minimum clinically significant difference for improvement is −3.38 points. Its validity and reliability have been established in patients with SSc.^21^^,^^24^^,^^25^

The HAMIS is a functional test to evaluate 4 specific movements consisting of flexion, extension, and abduction of the fingers and extension of the wrist. Various objects with standardized dimensions such as cutlery handles, pens, coffee bags, fishing line reels, milk cartons, and tables are used for this purpose. Using these objects, each movement of the hand is rated on a scale of 0-3. A score of 0 means a normal movement, while a score of 3 means that the hand cannot fully perform the movement in question. The total score for each hand is calculated based on the sum of the points obtained for each movement, with the highest score being 12.^26^

The DFTP is a test that measures the difference in centimeters between flexion and extension of the finger during finger movements to the hand. The lower the value, the smaller the range of motion.^27^

The patients who had completed the program at the end of the study (control group (n = 18), paraffin bath group (n = 17)) were included in the analysis. The intragroup changes were calculated by comparing the difference values of the patients at week 6 and week 12 with the baseline values. Both groups were then compared with each other.

### Statistical Analysis

The Shapiro–Wilk test was used to analyze the conformity of the continuous variables with the normal distribution. For continuous variables, the mean ± standard deviation or the median (Q1, Q3) was used, while n (%) was used for categorical variables. Variables that were not normally distributed were subjected to the Mann–Whitney *U-*test. For continuous variables with normal distribution, the independent samples *t*-test was used to compare 2 groups. The categorical variables were compared using the Fisher’s exact chi-square test. The Wilcoxon test was applied to dependent variables that weren’t distributed normally. The SPSS program version 23 (IBM SPSS Corp.; Armonk, NY, USA) was used to calculate the statistical data. The statistical significance level was *P* < .05.

## Results

**Figure 1.**
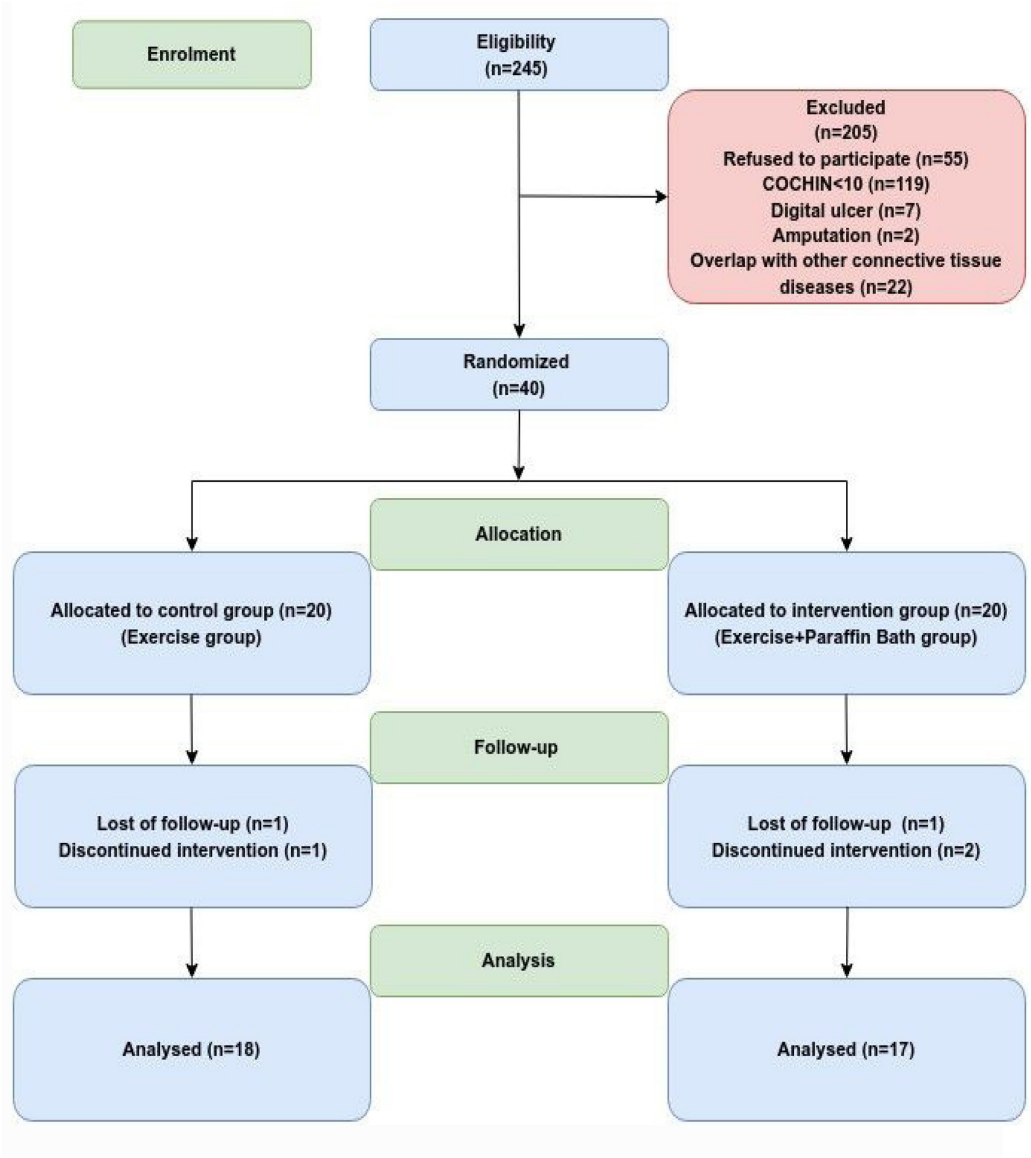
Flow diagram.

A total of 245 patients from the SSc cohort were screened for eligibility for the study (Figure 1). Thirty-five patients (control group (n = 18), paraffin bath group (n = 17)) who had completed the study period were evaluated. All patients who completed the study and were included in the analysis adhered to the exercise program.

### Clinical and Demographic Baseline Data of the Control Group and the Paraffin Bath Group

When the control group and the paraffin bath group were compared in terms of baseline characteristics, there was no statistically significant difference between the groups in terms of age, gender, duration of illness, medication used, and assessment parameters. Although the number of patients with limited SSc was higher in the paraffin group and the number of patients with diffuse SSc was higher in the control group, there was no statistically significant difference between the 2 groups (*P* = .407) (Table 1).

**Follow-Up Parameters of the Control Group And the Paraffin Bath Group 6 Weeks and 12 Weeks After Treatment**

When both groups were evaluated 6 and 12 weeks after treatment compared to baseline, there was a significant difference in the change scores of COCHIN, HAMIS-right hand, HAMIS-left hand, HGS-right hand, and HGS-left hand parameters in the control group at both the 6- and 12-week follow-up compared to baseline. In the paraffin bath group, there was a significant difference in the change values of the parameters NRS, COCHIN, DFTP-right hand, HAMIS-right hand, HAMIS-left hand, HGS-right hand and HGS-left hand after both 6 and 12 weeks (Table 2).

When comparing the 2 groups, there was a statistically significant difference in favor of the paraffin group in terms of the change in NRS scores at both the 6-week and 12-week follow-up, and the pain reduction was greater in the paraffin bath group. No difference was found in the other parameters (Table 2).

The patients had no side effects with either the exercises or the paraffin treatment.

## Discussion

As a result of the study, the hand therapeutic exercise program was found to significantly improve hand function in SSc patients, while the application of paraffin baths in addition to this program does not provide any additional benefit to hand function, but may provide further pain relief.

SSc remains a cause of mortality and morbidity, and a combination of pharmacologic and nonpharmacologic treatments is required for comprehensive management.^28^^,^^29^ Non-pharmacological treatments can improve quality of life and performance of daily activities by improving hand function and reducing pain.^30^ The EULAR has recently published nonpharmacological treatment recommendations for systemic lupus erythematosus and SSc, emphasizing that thermal procedures and hand exercise programs improve hand function in SSc patients.^5^

Although there are studies in the literature on hand exercise programs among non-pharmacological treatments,^4^^,^^6^^-^^13^ only 4 studies on paraffin bath applications among thermal modalities since the 2000s was found.^16^^-^^18^^,^^31^

In the study by Sandqvist et al^17^ from 2004, 17 patients were examined. One of the patients’ hands was treated with paraffin baths and exercise therapy for 1 month, while the other hand was treated with exercise therapy only. Improvements in certain parameters were observed in both groups and no significant difference was found between the groups. On the other hand, extensibility, perceived stiffness, and skin elasticity improved significantly more in the hand treated with paraffin than in the hand that was only exercised. In conclusion, hand flexibility can be improved by integrating paraffin baths into daily hand exercises. In the study, which is consistent with this study, there was an improvement in the parameters assessed in both groups compared with baseline values and there was no significant difference between the groups. On the other hand, the changes were greater in the paraffin group in terms of pain reduction and disability improvement.

A case series of 3 participants evaluated in 2009 by Mancuso et al^18^ examined the effect of paraffin baths and exercise on both improvement in impairment and participation in daily activities. The results highlighted the potential benefit of a physical modality combined with an active exercise program to improve hand function related to activity participation.

A randomized controlled trial published in 2019 with HAMIS as the primary outcome measure and 34 participants investigated the effect of adding paraffin bath therapy to a home exercise programme on hand function.^16^ During the study, the control group received an exercise program consisting of finger extension movements previously recommended by Mugi et al.^10^ In the intervention group, a paraffin bath was administered in addition to the exercises. At the end of the study, it was reported that the additional application of paraffin baths to the home exercises did not bring any additional benefit. On the other hand, only 1 participant discontinued the application due to pain after the exercise. The results of the study, which showed that paraffin bath application had no additional functional contribution to exercise, are consistent with this study. In addition, there were no participants who discontinued the study due to side effects, and obtained similar results in terms of safety. In contrast to this study, significant improvement in both groups was seen, particularly in the COCHIN measures. The most important factor here seems to be the use of a different protocol in the content of the exercise program, such as strengthening, finger and wrist movement exercises, as opposed to the finger stretching exercises in this study.

In another randomized controlled trial with 56 participants, published in 2019, the effects of warming the hands in warm water and warming them with paraffin before exercise on hand mobility and health status were compared. HAMIS scores improved in both groups. As a result of the study, it was found that warming with paraffin made no additional contribution to hand mobility compared to warm water before hand exercises.^31^ In this study, the application of paraffin and warm water were compared and there was no control group. On the other hand, although fewer patients (n = 90) were evaluated for eligibility, the number of patients included was higher than in the study. The most important factor here is that the included participants are in a better condition than the participants in the study, considering the baseline characteristics of the participants (HAMIS). Again in this study, the number and proportion of patients with limited and diffuse SSc differed between the 2 groups. The prognosis for patients with diffuse SSc may be worse than for those with limited SSc.^31^ In the study, the number of patients with limited SSc was higher in the paraffin group, while the number of patients with diffuse SSc was higher in the control group. This may introduce bias in the evaluation of patient outcomes. However, the scoring systems (NRS, COCHIN, DFTP-Right Hand, DFTP-Left Hand, HAMIS-Right Hand, HAMIS-Left Hand, HGS-Right Hand, HGS-Left Hand) used in the study primarily assess hand movements and functions rather than providing a systematic evaluation. Furthermore, the analysis showed that these differences were not statistically significant (*P* = .407).

The significant decrease in NRS scores in the paraffin bath group in the study is consistent with studies from the literature.^32^^-^^34^ When paraffin baths are used, the increase in local temperature, vasodilation, reduction in muscle cramps, and increased elasticity of connective tissue are the main factors contributing to this result.^35^

Pain in SSc patients may arise from various causes, such as vasospasm, finger ulcers, and joint contractures.^14^ These factors can impair hand function and quality of life. Reducing pain in the hands, which are frequently affected in SSc, can facilitate the implementation of exercises important for hand movement. Furthermore, paraffin bath therapy is a non-invasive method and may offer a way to avoid the potential side effects of non-steroidal anti-inflammatory drugs or other agents used to reduce pain in SSc patients, who are already undergoing numerous medical treatments and may also have gastrointestinal involvement.

The main limitation of the study is that although a total of 245 SSc patients were screened for eligibility, 35 patients could be included in the study. The main factors for this were the small number of patients with a COCHIN score ≥ 10 and significantly impaired hand function, and the refusal of patients to participate in the study. Another important limitation is that the participants could not be blinded to the study, as the treatments applied were exercises and paraffin baths. On the other hand, the NRS and COCHIN assessment forms were completed by the participants. In addition, the HAMIS, DFTP, and hand grip strength measurements at baseline and controls were performed by the same physiatrist blinded to the participants to minimize the risk of bias.

In summary, therapeutic hand exercises are effective in SSc patients with impaired hand function and should be part of the treatment. However, the addition of paraffin baths to the exercise program does not provide any additional benefit to hand function, but may be useful for pain relief.

## Figures and Tables

**Table 1.** Comparison of Baseline Clinical and Demographic Characteristics of Control and Paraffin Bath Groups

**Parameters**	**Control (n = 18)**	**Paraffin Bath (n = 17)**	***P***
Age (years)	53.2 ± 14.6	54.1 ± 9.7	.833^t^
Sex Female Male	17 (94.4%) 1 (5.6%)	15 (88.2%) 2 (11.8%)	.603^f^
Disease duration (years) Limited SSc Diffuse SSc	10.0 (3.5, 14.2) 6 (33.3%) 12 (66.7%)	9.0 (5.5, 12.0) 9 (52.9%) 8 (47.1%)	.909^m^ .407^y^ .407^y^
Medical therapies Glucocorticoids Methotrexate Azathioprine Cyclophosphamide Mycophenolate mofetil Rituximab NSAIDs Nifedipine Bosentan	8 (44.4%) 2 (11.1%) 2 (11.1%) 1 (5.5%) 8 (44.4%) 2 (11.1%) 2 (11.1%) 8 (44.4%) 2 (11.1%)	7 (41.2%) 3 (17.6%) 2 (11.8%) 0 (0.0%) 4 (23.5%) 3 (17.6%) 3 (17.6%) 7 (41.2%) 3 (17.6%)	1.000^y^ .658^f^ 1.000^f^ 1.000^f^ .344^y^ .658^f^ .658^f^ 1.000^y^ .658^f^
NRS	5.0 (3.5, 5.0)	5.0 (3.0, 7.0)	.832^m^
COCHIN	19.0 (11.0, 32.2)	29.0 (20.0, 42.50)	.126^m^
DFTP-right hand	8.0 (6.7, 9.5)	8.5 (6.5, 9.0)	.961^m^
DFTP-left hand	8.0 (7.0, 9.5)	8.5 (6.2, 9.5)	.909^m^
HAMIS-right hand	2.0 (0.7, 4.0)	2.0 (1.0, 4.5)	.590^m^
HAMIS-left hand	1.5 (0.0, 3.0)	2.0 (1.0, 4.5)	.463^m^
HGS-right hand	19.4 ±7.6	18.9 ± 6.9	.835^t^
HGS-left hand	19.7 ± 6.7	17.6 ± 5.4	.305^t^

Quantitative variables presented as mean ± standard deviation or median (Q1-Q3) values; qualitative variables presented with n (%).

COCHIN, Cochin Hand Functional Scale; DFTP, Delta finger-to-palm; HAMIS, modified hand mobility in Scleroderma; HGS, hand grip strength; NSAIDs, non-steroidal anti-inflammatory drugs; NRS, Numerical Rating Scale; SSc, systemic sclerosis; *P* < .05: statistical significance level; f, Fisher’s exact test; m, Mann–Whitney *U-*test; t, independent samples *t* test; χ², chi-square test; y, Yates chi-square test.

**Table 2.** Comparison of the Control and Paraffin Bath Groups in Terms Of Follow-up Parameters and Changes At 6 Weeks And 12 Weeks After Treatment

	**6 weeks**			**12 weeks**		
**Parameters**	**Control** **(n = 18)**	**Paraffin Bath (n = 17)**	***P***	**Control** **(n = 18)**	**Paraffin Bath (n = 17)**	***P***
NRS Before-after treatment change Change within group *P*	4.1 ± 2.3 0.0 (−1.0, 1.0) .463^w^	3.1 ± 2.3 −2.0 (−4.0, −1.0) **.014** **^w^**	.256^t^ **.007** **^m^**	5.0 (2.0, 5.0) 0.0 (−0.2, 0.0) .380^w^	3.0 (1.0, 5.0) −2.0 (-3.0, −1.0) **.002** **^w^**	.134^m^ **<.001** **^m^**
COCHIN Before-after treatment change Change within group *P*	11.5 (3.7, 22.5) −8.0 (−10.2, −5.0) **<.001** **^w^**	17.0 (11.5, 23.0) −10.0 (−15.0, −5.0) **<.001** **^w^**	.268^m^ .362^ m^	11.5 (4.7, 22.2) −7.0 (−9.2, −5.0) **<.001** **^w^**	18.0 (14.0, 24.0) −9.0 (−16.0, −4.5) **<.001** **^w^**	.290^m^ .296^ m^
DFTP-right hand Before-after treatment change Change within group *P*	7.7 (7.0, 9.5) 0.0 (0.0, 0.1) .339^w^	9.0 (6.7, 9.3) 0.5 (0.0, 0.5) **.026** **^w^**	.855^m^ .146^m^	7.7 (7.0, 9.5) 0.0 (0.0, 0.1) .340^w^	9.0 (6.7, 9.3) 0.5 (0.0, 0.5) **.036** **^w^**	.921^m^ .232^m^
DFTP-left hand Before-after treatment change Change within group *P*	7.7 (7.3, 9.7) 0.0 (0.0, 0.1) .603^w^	8.5 (6.8, 9.7) 0.0 (0.0, 0.5) .190^w^	>.05^m^ .346^m^	7.7 (7.3, 9.7) 0.0 (0.0, 0.1) .546^w^	8.5 (7.5, 9.5) 0.0 (0.0, 0.5) .271^w^	.934^m^ .727^m^
HAMIS-right hand Before-after treatment change Change within group *P*	1.0 (0.0, 2.5) 0.0 (−1.2, 0.0) **.011** **^w^**	2.0 (1.0, 3.0) 0.0 (−1.0, 0.0) **.042** **^w^**	.223^m^ .434^m^	1.0 (0.0, 2.5) 0.0 (−1.2, 0.0) **.010** **^w^**	2.0 (1.0, 3.0) 0.0 (−1.0, 0.0) **.041** **^w^**	.236^m^ .541^m^
HAMIS-left hand Before-after treatment change Change within group *P*	1.0 (0.0,2.0) 0.0 (−1.2, 0.0) **.011** **^w^**	2.0 (0.5, 3.0) 0.0 (-1.0, 0.0) **.041** **^w^**	.235^m^ .480^m^	1.0 (0.0, 2.0) 0.0 (−1.2, 0.0) **.010** **^w^**	2.0 (0.5, 3.0) 0.0 (−1.0, 0.0) **.038** **^w^**	.255^m^ .566^m^
HGS-right hand Before-after treatment change Change within group *P*	21.4 ± 8.4 1.5 (0.9, 3.4) **.002** **^w^**	22.0 ± 7.2 2.3 (1.0, 5.0) **.006** **^w^**	.829^t^ .389^m^	21.6 ± 8.8 1.5 (0.5, 3.2) **.003** **^w^**	21.3 ± 6.8 2.0 (0.5, 3.9) **.003** **^w^**	.931^t^ .817^m^
HGS-left hand Before-after treatment change Change within group *P*	21.1 ± 7.8 1.5 (0.6, 2.7) **.011** **^w^**	20.6 ± 6.3 2.0 (1.0, 4.0) **.001** **^w^**	.830^t^ .137^m^	21.5 ± 8.6 1.3 (0.6, 2.0) **.018** **^w^**	19.6 ± 6.0 1.3 (0.3, 2.5) **.001** **^w^**	.465^t^ .704^m^

Quantitative variables presented as mean ± standard deviation or median (Q1, Q3) values; qualitative variables presented with n (%).

COCHIN, Cochin Hand Functional Scale; DFTP, Delta finger-to-palm; HAMIS, modified hand mobility in Scleroderma; HGS, hand grip strength; NRS, Numerical Rating Scale; *P* < .05: statistical significance level; m, Mann–Whitney *U-*Test; t, independent samples* t* test; w, Wilcoxon test.

^w,m^The statistical significance of the change in the parameters in the control and paraffin groups at baseline and at weeks 6 and 12.

## Data Availability

The data that support the findings of this study are available on request from the corresponding author.
